# The Auto-Inhibitory Role of the EPAC Hinge Helix as Mapped by NMR

**DOI:** 10.1371/journal.pone.0048707

**Published:** 2012-11-21

**Authors:** Rajeevan Selvaratnam, Mohammad T. Mazhab-Jafari, Rahul Das, Giuseppe Melacini

**Affiliations:** 1 Department of Chemistry and Chemical Biology, McMaster University, Hamilton, Ontario, Canada; 2 Department of Biochemistry and Biomedical Sciences, McMaster University, Hamilton, Ontario, Canada; Griffith University, Australia

## Abstract

The cyclic-AMP binding domain (CBD) is the central regulatory unit of exchange proteins activated by cAMP (EPAC). The CBD maintains EPAC in a state of auto-inhibition in the absence of the allosteric effector, cAMP. When cAMP binds to the CBD such auto-inhibition is released, leading to EPAC activation. It has been shown that a key feature of such cAMP-dependent activation process is the partial destabilization of a structurally conserved hinge helix at the C-terminus of the CBD. However, the role of this helix in auto-inhibition is currently not fully understood. Here we utilize a series of progressive deletion mutants that mimic the hinge helix destabilization caused by cAMP to show that such helix is also a pivotal auto-inhibitory element of apo-EPAC. The effect of the deletion mutations on the auto-inhibitory apo/inactive *vs.* apo/active equilibrium was evaluated using recently developed NMR chemical shift projection and covariance analysis methods. Our results show that, even in the absence of cAMP, the C-terminal region of the hinge helix is tightly coupled to other conserved allosteric structural elements of the CBD and perturbations that destabilize the hinge helix shift the auto-inhibitory equilibrium toward the apo/active conformations. These findings explain the apparently counterintuitive observation that cAMP binds more tightly to shorter than longer EPAC constructs. These results are relevant for CBDs in general and rationalize why substrates sensitize CBD-containing systems to cAMP. Furthermore, the NMR analyses presented here are expected to be generally useful to quantitatively evaluate how mutations affect conformational equilibria.

## Introduction

**Figure 1:**
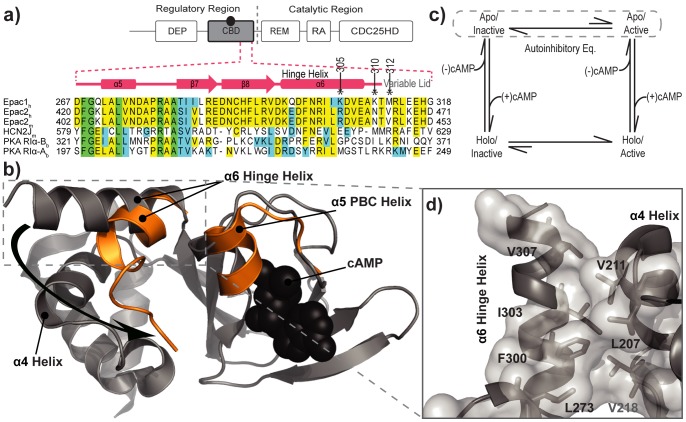
The CBD of EPAC and the domain organization. **a**) The regulatory region consists of the DEP (disheveled Egl-10 pleckstrin) domain and the cAMP binding domain (CBD), colored grey. The catalytic region includes the CDC25 homology domain (CDC25HD), the Ras exchange motif (REM), and Ras association (RA) domain. The dashed red lines illustrate an expanded view of the sequence alignment of CBDs for the regions spanning the PBC α5 helix to the Hinge Helix. The corresponding secondary structure is shown above the sequence. The asterisks mark the site of termination in the deletion mutants. **b**) The structure of the CBD of apo-EPAC is shown in grey, whereas the major changes caused by cAMP (black spheres) binding are shown in orange. The curved black arrow illustrates the transition of the hinge helix from the apo form (grey; PDB ID: 2BYV) to the holo form (orange; PDB ID:3CF6). **c**)The thermodynamic cycle of cAMP dependent EPAC activation. Dashed lines encircle the equlibrium between the apo/inactive and apo/active states, *i.e.* the auto-inhibitory equilibrium. **d**) The hydrophobic “spine”, a network of residues involving the hydrophobic contacts between the hinge helix and adjacent helices (α4 and α5).

The cAMP binding domain (CBD) is an ancient regulatory module found throughout multiple proteins with diverse functions [1]–[3]. For example, in prokaryotes, a CBD is present in the transcription factor, catabolite activator protein (CAP) [4], [5]. In eukaryotes, CBDs are found in Protein Kinase A and G [1], [2], [6]–[18], in transport proteins, hyperpolarization activated and cyclic-nucleotide modulated (HCN) channels [19], [20], as well as in the guanine nucleotide exchange factors, EPAC (Fig. 1) [3], [10], [21]–[28]. Although, these aforementioned proteins are functionally diverse, the embedded CBD(s) play a similar allosteric role – regulation by means of auto-inhibition [29], [30], *i.e.* the CBDs maintain a state of inactivity in the absence of the endogenous agonist, cyclic-AMP (cAMP) [22], [23], [25], [27], [31], [32]. Binding of cAMP acts by releasing the inhibition exerted by the auto-inhibiting determinants of the CBDs.

The CBDs are typically characterized by an eight stranded jelly-roll β-sandwich, flanked by helices at the N- and C-termini as well as a small intervening helix situated between strands β6 and β7 (Fig. 1A, 1B) [1], [2]. Recent methods aimed at comparing patterns of amino acid conservations in sequence [1] and in space [2] have identified four conserved structural elements that are universally present in eukaryotic CBDs: the N-terminal helical bundle, the β2-β3 loop, the phosphate binding cassette (PBC) and the hinge helix [2]. Previous investigations on the CBD of EPAC1, have established the former three structural elements as crucial determinants underlying auto-inhibition [10], [21], [27]. However, the role of the hinge helix as an auto-inhibitory determinant of the EPAC CBD is currently not fully understood.

The last two turns of the EPAC hinge helix (called α6, Fig. 1A) partially unfold as α6 rotates towards the α5 helix of the PBC upon cAMP binding (Fig. 1B) [21], [28], [33]. This hinge rotation has been rationalized as a consequence of the cAMP-induced repositioning of the PBC L273 residue, which contacts with F300 in the hinge helix. The repositioning of the conserved L273, and consequently F300, retracts the hinge helix toward the PBC helix upon activation (Fig. 1B) [23], [25], [27], [31].

Recent studies mapping the EPAC allosteric network through chemical shift covariance analysis (CHESCA) have revealed that L273 and F300 are part of a larger cluster of allosteric residues, which includes also a hydrophobic spine at the interface between the α4 and α6 helices (Fig. 1D) [26]. Such spine spans residues in the C-terminal end of the hinge helix that unwinds upon cAMP binding (*i.e.* 305–310, Fig. 1D).

Based on these observations, here we hypothesize that the C-terminal residues of the hinge helix (*i.e.* residues 305–310) are key determinants of EPAC auto-inhibition and that perturbations that destabilize the helix or induce unwinding shift the apo/inactive *vs.* apo/active pre-equilibrium toward the latter state, *i.e.* an active state without cAMP (Fig. 1C). To test this hypothesis, we designed three successive deletion mutations of the 149–318 EPAC1 construct [10], [21], which spans the CBD and which from here on forth will be referred to as the Wt-EPAC. Specifically, these mutants are C-terminally truncated at positions 305, 310, and 312 (called de305, de310, and de312, respectively from here on forth) and act as perturbations that destabilize the hinge helix of apo-EPAC, mimicking the cAMP-induced unwinding (Fig. 1B).

**Figure 2:**
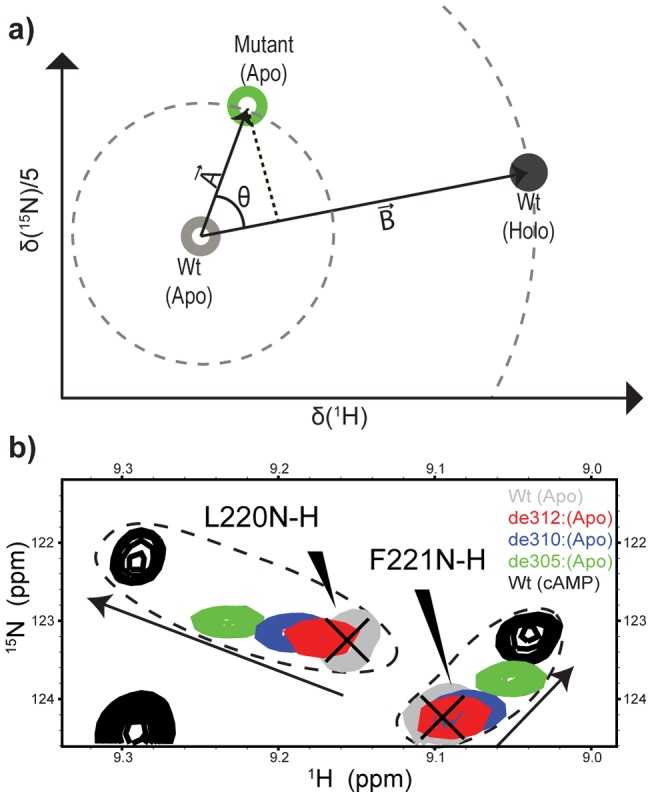
Chemical shift projection analysis (CHESPA) using mutations as perturbations. **a**) Schematic of CHESPA. Open circles indicate HSQC peaks of the apo forms, whereas the filled circle represents the holo form (cAMP bound) HSQC peak. The green open circle represents the apo-mutant. The compounded chemical shift between the Wt(apo) and Wt(holo) was computed as the magnitude of the vector B, |B|. Similarly the compounded chemical shift between the Wt(apo) and Mutant(apo) was calculated as |A|. The magnitude of vectors A and B define the radii of the dashed circles centered on the Wt(apo) peak (**b**) Representative regions of the [^15^N-^1^H] HSQC spectra of Wt(apo) (grey) and cAMP-bound, Wt(holo) (black) overlaid with the [^15^N-^1^H] HSQC spectra of apo-Mutants: de312 (red), de310 (blue), de305 (green). Arrows indicate the direction of shift toward activation and dashed contour lines enclose peaks of the same residues.

In order to explore how the mutations affect the inactive *vs.* active conformational equilibrium of apo-EPAC, we employed the previously proposed chemical shift projection analysis (CHESPA), which provides residue-specific fractional shift towards activation for each mutant (Fig. 2A) [27]. In addition, the allosteric role of the hinge helix was further probed by the chemical shift covariance analysis (CHESCA), for which mutations were utilized as source of perturbations, unlike in previous CHESCA applications where cAMP and analogs were used to perturb the allosteric system [26].

Our results confirm the hypothesis that the C-terminal residues of the hinge helix (*i.e.* residues 305–310) are a pivotal determinant of EPAC auto-inhibition, showing that the hinge helix is extensively coupled to the other conserved allosteric elements of the CBD, even in the absence of cAMP. These results also lead to the counter-intuitive prediction that deletion of this C-terminal region causes an enhancement in cAMP-affinity, due to an increase in the apo/active relative population. This unexpected prediction was corroborated by the measurement of cAMP-binding isotherms through saturation transfer difference (STD) NMR experiments and the relevance of these results for the substrate-dependent sensitization to cAMP is also discussed [34], [35].

## Materials and Methods

### Sample preparation

The deletion mutations de312, de310, and de305 were generated by inserting a stop codon at position 313, 311, and 306, respectively, by PCR in the Wt construct (EPAC1_149–318_) and confirmed by DNA sequencing. Wt and all mutant constructs including E308A were purified and labelled according to published methods [26].

### NMR Measurements

Spectra were acquired with a Bruker Avance 700-MHz spectrometer equipped with a 5 mm TCI cryoprobe at 306 K. Gradient and sensitivity enhanced [^1^H-^15^N] heteronuclear single quantum coherence (HSQC) were recorded for a total of 8 scans per t1 point. The number of digitized complex points were 256 and 1024 for the ^15^N and ^1^H dimensions, respectively, with an inter-scan delay of 1 sec. Carrier frequencies of the ^15^N and ^1^H channels were centered on water and the backbone amide region, respectively. All spectra were processed using NMRPipe [36] with linear prediction and a resolution-enhancing 60° shifted squared sine bell window function for HSQC spectra. Cross-peaks were assigned and integrated using Gaussian line-fitting in SPARKY [37]. Assignments were obtained using triple-resonance experiments [21], [38]. All samples were referenced using the internal referencing compound ^15^N-Ac-Glycine.

### Chemical Shift Projection Analysis (CHESPA)

The projection analysis descriptors, *i.e.* the cos Θ values, the fractional activations X and the compounded chemical shift differences between the apo-Wt and the apo-mutants (Fig. 2A) were computed as previously described [27]. In brief, the compounded chemical shift difference between the apo-Wt and the apo-mutants was calculated as the magnitude of vector A in Figure 2A. Similarly, the compounded chemical shift difference between the Wt(apo) and the Wt(holo) was calculated as the magnitude of the activation vector B in Figure 2A. The chemical shift (ppm) of the ^15^N was downscaled by a factor of 0.2, as indicated in Figure 2A. The cos Θ and fractional activation X were calculated as:
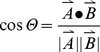
(1)

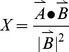
(2)


### Chemical Shift Covariance Analysis (CHESCA)

The inter-residue correlation matrix was generated according to published protocols [26]. However, in contrast to previous applications of CHESCA, the perturbation set was composed of select mutations that destabilize the C-terminal end of the hinge helix. Such mutations were analyzed in the apo state, where the extended hinge helix is stable. Thus, the perturbation set used here for CHESCA consisted of: Wt(apo), de305(apo), de310(apo), de312(apo) and E308A(apo).

### Singular Value Decomposition (SVD) Analysis of Deletion Mutants

The SVD analysis is based on previously published protocols [26], which were adapted and extended here for the application to deletion mutants. Specifically, a matrix **M** containing the combined chemical shifts for each assigned residue was first generated for five selected states: apo-Wt, cAMP-bound Wt, Sp-cAMPS-bound Wt, Rp-cAMPS-bound Wt and a 5th state that consisted of one of the deletion mutants in the apo form (*i.e*. de312, de310 or de310) or the apo-L273W. The combined chemical shifts (δ_NH_) were calculated as δ_NH_  = 0.2δ_N_ + δ_H_, where δ_N_ and δ_H_ are the individual chemical shift (ppm) values of the backbone ^15^N and ^1^H nuclei [26], [39]. Only residues for which the frequency spread across all five states was greater than 5 and 10 Hz for the individual ^1^H and ^15^N nuclei, respectively, were considered. A matrix **M'** was then subsequently generated from **M** in which the Rp-cAMPS-bound Wt state was used as reference for the remaining four states. Specifically, the columns of the **M’** matrix were: Wt(apo)–Wt(Rp-cAMPS), Wt(cAMP)–Wt(Rp-cAMPS), Wt(Sp-cAMPS)–Wt(Rp-cAMPS) and a 4th state with a deletion mutant or L273W in the apo form measured relative to Wt(Rp-cAMPS) (*i.e.* de312(apo)–Wt(Rp-cAMPS), de310(apo)–Wt(Rp-cAMPS), de305(apo)–Wt(Rp-cAMPS) or L273W(apo)–Wt(Rp-cAMPS)). The matrix **M'** was then column mean centered and factorized through SVD as previously explained [26]. The first two principal components (PCs) resulting from the SVD analyses performed here account for >93% of the total variance (Table 1) and therefore the other PCs were deemed negligible and discarded.

**Table 1:** Total Variance Breakdown in the SVD Analysis of Deletion Mutants and L273W.

Mutant	Principal Components (PCs)	Percentage of Total Variance
EPAC_149–312_ (de312)	PC1	52.4%
	PC2	44.8% (97.2%)*
EPAC_149–310_ (de310)	PC1	59.8%
	PC2	36.3% (96.1%)*
EPAC_149–305_ (de305)	PC1	57.3%
	PC2	35.8% (93.1%)*
EPAC_149–318_ (L273W)	PC1	72.3%
	PC2	23.4% (95.7%)

* The percentages reported in parentheses are the cumulative contribution of PC1 and PC2 for each SVD analysis involving a mutant.

### cAMP Binding Measurements

The dissociation constant (K_D_) for cAMP from Wt and de305 were measured through the saturation transfer difference (STD) amplification factor (STDaf) [24], [40]. All STD measurements were carried out with a solution of 25 μM of Wt or 15 μM de305 in 20 mM phosphate buffer, pH 7.6, 50 mM NaCl, 99.9% D_2_O and at 25°C. The 1D-STD spectra were acquired at total cAMP concentrations of 25, 50, 75, 100, 150, 200 and 300 μM [24]. Separate reference 1D (STR) experiments were also acquired. The STD amplification factor (STDaf) was calculated as the product of the STD/STR ratio (measured for the well resolved cAMP ribose H1' at 6.2 ppm) and of the ratio of the total cAMP and protein concentrations. The STDaf values were then normalized relative the STDaf plateau value reached at high cAMP concentrations ([cAMP]_Tot._ ≥150 μM). The normalized STDaf values were then analyzed with the binding isotherm equation: Normalized STDaf  = 1– (1/ (1+ ([cAMP]/K_D_))), where [cAMP] is the concentration of free cAMP [24], [40].

## Results and Discussion

### CHESPA analysis of de305, de310 and de312

**Figure 3:**
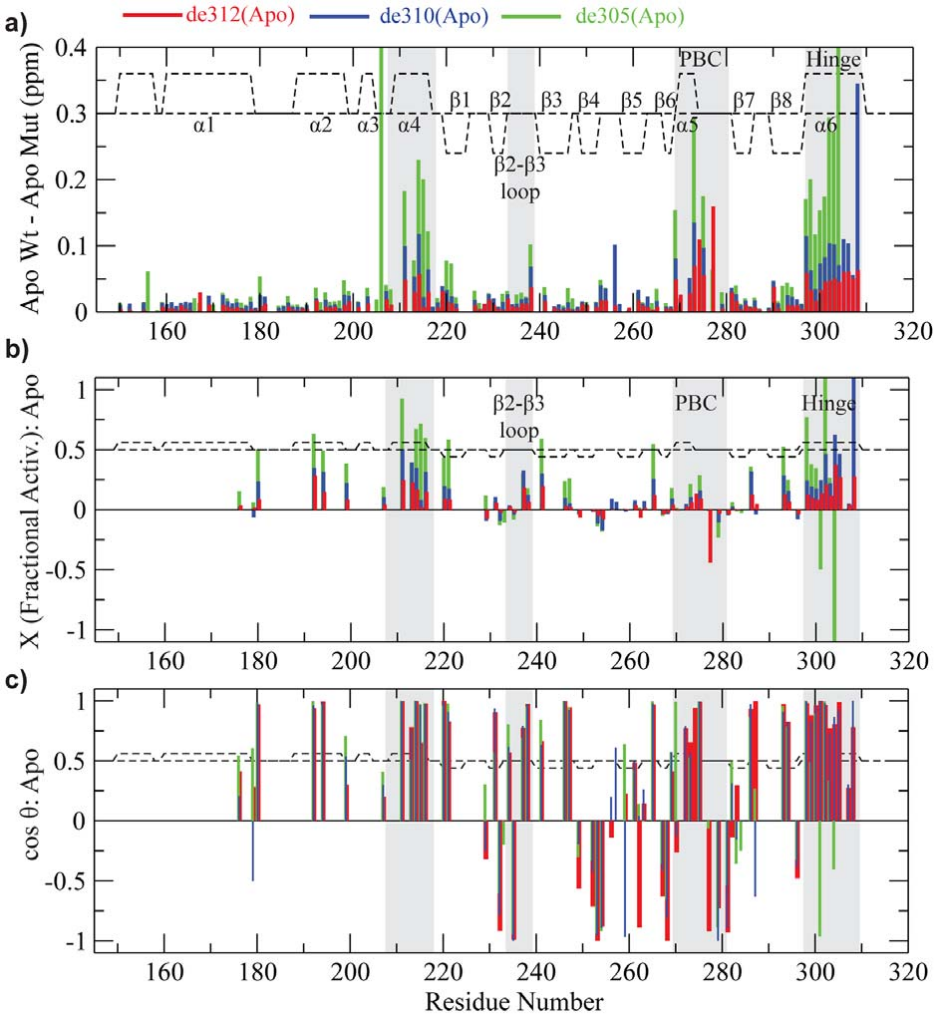
Chemical shift projection analysis to map the effects of the apo truncation mutants de312 (red), de310 (blue) and de305 (green) relative to Wt(apo). The dashed lines represent the secondary structure of the apo-EPAC (PDB ID: 2BYV). The grey highlights are regions subject to some of the most significant cAMP-dependent changes on the Wt(apo). (**a**) The compounded chemical shift profile of the apo-mutants relative to apo-Wt, that is the magnitude of vector A in Figure 2A. (**b**) Fractional shift toward activation achieved by the mutations in the absence of cAMP and with compounded chemical shifts greater than 0.05 ppm between the Wt(apo) and Wt(holo) state. (**c**) Cosine values for the projection angle, as in Figure 2A, which is also an indicator of the direction of chemical shift movement along the activation path (vector B in Fig. 2A).

To investigate the effects of the C-terminal deletion mutations, we purified and assigned de305, de310 and de312 in the apo states and compared them to the Wt(apo) and cAMP-bound states (Fig. 2A). We first analyzed the de312 truncation mutant (*i.e.* EPAC1_149–312_), which leaves the hinge region (residues 296–310) to a large extent intact but removes the C-terminal tail of the Wt construct, EPAC1_149–318_. The residue profile of the compounded chemical shift differences between Wt(apo) and de312(apo) (Figure 3A, red bars) exhibits local maxima in the regions most affected by cAMP-binding (Fig. 3A, grey regions) [9], [21]. In addition, the [^15^N-^1^H]-HSQC spectral comparison of the de312(apo) mutant relative to the Wt(apo) and cAMP-bound states for well dispersed and isolated peaks (Fig. 2B) reveals a slight but consistent shift for de312 towards the active state. However, in order to systematically assess at residue resolution the effect of the de312 mutation on the apo/inactive *vs.* apo/active auto-inhibitory equilibrium, we took advantage of the recently developed chemical shift projection analysis (CHESPA) (Fig. 2A; Fig. 3B, 3C, red bars). While the compounded chemical shifts quantify only the size of the perturbation, the fractional activation X obtained from the projection analysis (Fig. 3B) together with the cosine Θ values (Fig. 3C) reflect both the direction and extent of the mutational perturbation toward the apo/active state.

The fractional shifts obtained though the projection analysis reflect four main effects: (a) nearest neighbour effects experienced by residues in close spatial proximity to the site of the mutation; (b) mutation specific perturbations on interaction networks that involve the mutated site; (c) nearest neighbour effects experienced by residues in the binding site for the endogenous allosteric effector, *i.e.* cAMP in our case, as we use the Wt(apo) and Wt cAMP-bound (holo) states to define vector B (Fig. 2A); (d) changes in the inactive *vs.* active two-state equilibrium caused by the mutation (examined here for the apo samples). The projection analysis presented here is aimed at isolating the residues that reflect mainly effect (d). Effect (d) is residue independent, but effects (a-c) lead to residue-dependent variations in the fractional shifts. The effect (d) is best represented by the fractional activation (X) measured for the residue with cosine Θ absolute values ∼1 (Figure 3C). In the case of de312(apo), the majority of such residues exhibit positive fractional activation values (Fig. 3B, red bars). These regions are also subject to the largest chemical shift changes caused by cAMP (Fig. 3, grey zones)[10], [21], suggesting de312(apo) mutation shifts the pre-equilibrium toward apo/active conformations.

The CHESPA analysis of de310(apo) and de305(apo) mutants leads to results similar to those obtained for de312(apo), but with overall larger chemical shift differences and fractional activation values (Figure 3A–C), indicating that these mutations further destabilize the C-terminal hinge helix. The de310(apo) and de305(apo) constructs appear therefore to mimic the apo/active state more closely than de312(apo). However, due to structural distortions introduced by these mutations, the fractional activation values appear to be somewhat residue dependent (Fig. 3B) and based on the projection analysis alone it is not possible to obtain a reliable quantitative estimate of the overall relative shift towards the active state caused by the C-terminal truncation. In order to circumvent this limitation of the projection analysis, we utilized a recently introduced alternative approach based on singular value decomposition (SVD) of NMR chemical shifts [26], which provides an improved isolation of the ppm changes that exclusively reflect variations in the position of the inactive *vs.* active equilibrium.

### The Singular Value Decomposition (SVD) analysis of the C-terminal truncation mutant indicates that the hinge helix residues 305–310 contribute to auto-inhibition

**Figure 4:**
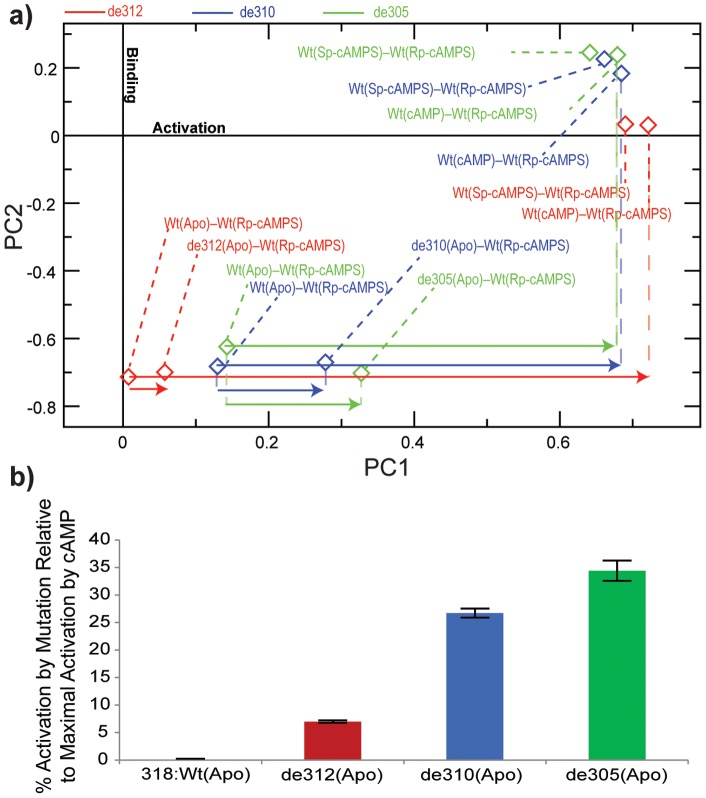
SVD analysis of the chemical shifts measured for the C-terminal truncation mutants de305, de310 and de312. **a**) This panel shows the PC1 vs. PC2 plot with three sets of loadings (diamonds) for each of the C-terminal hinge helix deletion mutants: de312 (red), de310 (blue) and de305 (green). There are four loadings per mutant with each loading corresponding to a state referenced to Rp-cAMPS, as labelled in the figure. The smaller arrows correspond to the separation along PC1 between the Wt(apo) and the mutant(apo) state. The large arrows correspond to the separation along PC1 between the Wt(apo) and the cAMP-bound Wt(holo). **b**) The percentage ratio of the two separations measured in panel (a) (*i.e.* relative magnitude of the two arrows), provides a quantitative measure of the overall fractional shift toward activation caused by the mutation.

In the previously outlined SVD analysis of chemical shifts [26], HSQC spectra for the Wt EPAC1 construct were acquired and assigned in five different states: the Wt(apo) as well as four Wt-bound states, saturated with cAMP, Sp-cAMPS, 2’-OMe-cAMP and Rp-cAMPS. The Sp-cAMPS and 2’-OMe-cAMP analogs are both EPAC activators, while Rp-cAMPS functions as an EPAC antagonist, *i.e.* it binds the EPAC1 CBD without causing activation and is therefore used as a chemical shift reference state in the SVD protocol [26]. Here, we use a similar SVD analysis, but we replace the 2’-OMe-cAMP-bound state with one of the mutants under investigation, *e.g.* the de312(apo) mutant. The 2’-OMe-cAMP was selected for this replacement because two other activators are already included in the analysis (*i.e.* cAMP and Sp-cAMPS) and therefore the SVD analysis is meaningful even in the absence of the 2’-OMe-cAMP state. Through this approach, the projection analysis is effectively expanded to include not only the Wt(apo) and cAMP-bound reference states (Fig. 2), but also the Sp-cAMPS- and Rp-cAMPS-bound forms, leading to an improved identification of the chemical shift changes that reflect uniquely variations in the activation equilibrium. For instance, when the 2’-OMe-cAMP-saturated state is replaced with the de312(apo) mutant, the first two principal components (PC) computed through SVD (*i.e.* PC1 and PC2) account for more than 93% of the total variance (Table 1). PC1 reflects activation whereas PC2 is reflective of binding effects, as illustrated in Figure 4A by the Wt(Sp-cAMPS)–Wt(Rp-cAMPS) and Wt(cAMP)–Wt(Rp-cAMPS) loadings aligned with PC1 and the Wt(apo)–Wt(Rp-cAMPS) loading aligned with PC2. The PC1 component of the difference between the Wt(cAMP)–Wt(Rp-cAMPS) and the Wt(apo)–Wt(Rp-cAMPS) loadings provides therefore a measure of the maximal activation caused by cAMP and is utilized to normalize the PC1 component of the difference between the mutant(apo)–Wt(Rp-cAMPS) and the Wt(apo)–Wt(Rp-cAMPS) loadings (Fig. 4A, red arrows). This ratio of these PC1 components indicates that the de312(apo) deletion mutant causes a 7% shift towards the apo/active conformers (Fig. 4B). The reliability of this approach was cross-validated by applying the SVD method to L273W (Figure S1 in Supporting Information), which leads to a 47% shift of the Wt(apo) equilibrium towards the inactive conformers, consistent with previous analyses [27]. A similar approach was also used to analyze the other two C-terminal deletion mutants, *i.e.* de310 and de305 (Fig. 4A, blue and green symbols, respectively), which cause further destabilization of the α6 helix. The percentage shifts towards activation caused by the successively truncating mutations de312, de310 and de305 are summarized in Figure 4B. Figure 4B shows that the de310 and de305 truncations result in a further dramatic increase in the relative population of the apo/active conformers to 27% and 35%, respectively. Overall, the SVD analyses of Figure 4A indicate that, while deletion of the C-terminal tail in de312 causes only a subtle shift towards activation, perturbations in the C-terminal region of the hinge helix, implemented through the de310 and de305 truncations, lead to a more drastic stabilization of the active conformation in the absence of cAMP. These results are in agreement with the overall findings of CHESPA (Fig. 3B, 3C) and together consistently point to a significant and previously unanticipated auto-inhibitory role for residues 305–310 of the EPAC hinge helix.

### The covariance analysis of chemical shifts reveals that the hinge-helix is coupled to the whole allosteric network of the EPAC CBD

**Figure 5:**
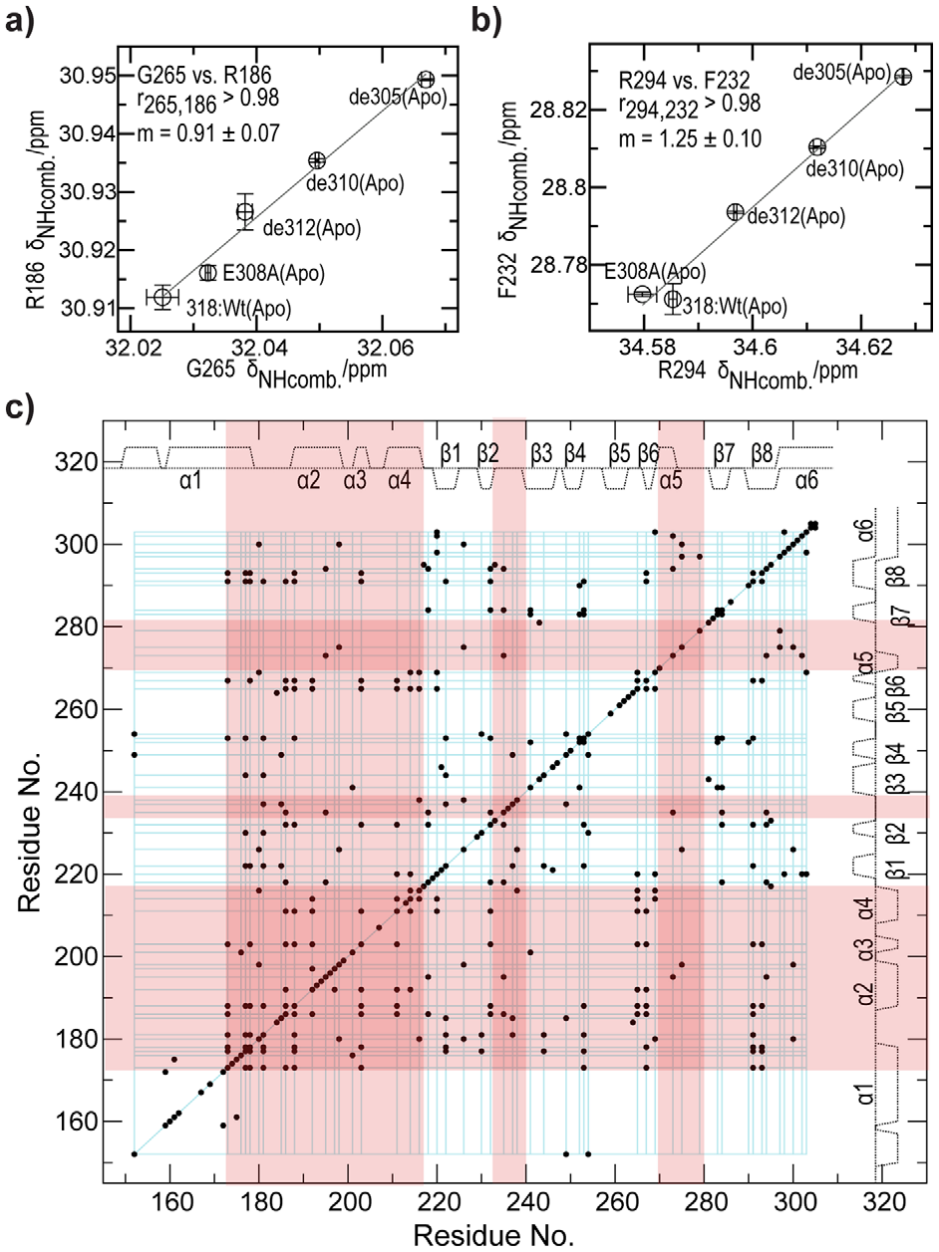
Chemical shift covariance analysis (CHESCA) of the hinge helix mutants. **a**) and **b**) show representative inter-residue chemical shift correlation among the five apo states (318:Wt, 318:E308A, de312, de310, and de305) and ‘m’ defines the slope. **c)** The chemical shift correlation matrix. Residue pairs with absolute correlation coefficients ≥0.98 are marked with a dot. The blue grid represents the largest agglomerative cluster (Figure S2 in Supporting Information) [26], while regions highlighted in red correspond to key allosteric sites of the CBD other than the hinge helix.

In order to further explore the allosteric network controlled by residue 305–310 of EPAC1 in the absence of cAMP, we implemented the chemical shift covariance analysis (CHESCA) method [26] using as basis set the Wt(apo), de312(apo), de310(apo) and the de305(apo) truncation mutants as well as E308A(apo), which also targets the 305–310 regions. Using these five apo EPAC1 samples, several linear inter-residue chemical shift correlations are observed (Fig. 5A, 5B), resulting in a residue correlation matrix (Fig. 5C) that reveals the presence of an extensive long-range network of interactions controlled by the 305–310 α6 region. Specifically, the agglomerative cluster analysis (Figure S2 in Supporting Information) of the correlation matrix (blue grid, Fig. 5C) indicates that perturbations on residues 305–310 propagate to all the known allosteric sites of the EPAC1 CBD, from the PBC and the β2-β3 loop to most of the N-terminal helical bundle (red highlights, Fig. 5C). Based on these observations, we conclude that the unwinding of residues 305–310 in α6 is coupled to the whole allosteric network controlled by cAMP (Fig. 5C).

### Destabilization of the hinge helix enhances the affinity for cAMP

**Figure 6:**
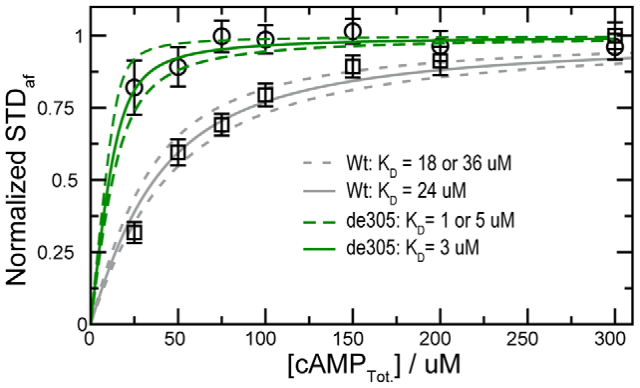
Binding isotherms for the titration of cAMP into an NMR sample with 15 **µM de305 (green) and 25** **µM Wt (grey) in 20** **mM phosphate buffer, pH** **7.6, 50** **mM NaCl, 99.9% D_2_O, and at 25°C.** The binding of cAMP to de305 and Wt was monitored through the STD amplification factor (STDaf) normalized to the plateau value and plotted versus the total cAMP concentration. The binding of cAMP to the Wt construct, Epac1_149–318_ was measured here to ensure an unbiased comparison to de305 since previous measurements [22], [34] were on Epac1_149–317_ and used different experimental conditions and methods.

Considering that the apo/active state binds cAMP more tightly than the apo/inactive state, the coupling between the C-terminal region of α6 revealed by the combined CHESPA and CHESCA methods, leads to the interesting prediction that de305, the closest mimetic of the apo/active form in our current investigation of the hinge helix (Fig. 4B), should exhibit higher affinity for cAMP than the Wt construct. This counter-intuitive prediction was experimentally confirmed by STD NMR measurements on both the de305 and the Wt construct (Fig. 6). As expected, Figure 6 clearly shows that the de305 mutant binds cAMP more tightly than Wt CBD with the full integral hinge helix. The ∼8-fold decrease in K_D_ observed in going from the Wt to the de305 mutant rationalizes the observation that substrates sensitize CBDs to cAMP [34], [35]. Substrates promote the open (active) topology of EPAC and consequently the unwinding of the hinge helix in apo-EPAC, which in turn results in higher affinity for cAMP, explaining the lower K_D_ value measured for the dissociation of cAMP from EPAC in the presence of the Rap substrate [34]. Interestingly, such sensitization of the CBD for cAMP in the presence of a substrate has also been observed for the CBD of PKA type I [35], suggesting that the auto-inhibitory role revealed here for the hinge-helix of EPAC may be relevant also for other cAMP-dependent systems.

## Conclusions

The hinge helix is a universally conserved structural element of the CBDs. Here we have shown that in the CBD of EPAC, the C-terminal region of the hinge helix is an important determinant of auto-inhibition and is tightly coupled to the other conserved allosteric CBD elements even prior to cAMP binding. Alleviating the contributions of the hinge helix to auto-inhibition, as engineered for example through mutations, favours the active conformations even in the absence of the cAMP allosteric effector and consequently enhances the affinity of the CBD for cAMP. Overall, these results are relevant for CBDs in general and explain why substrates sensitize CBD-regulated systems to cAMP [34], [35]. Furthermore, the NMR analyses presented here are expected to be generally useful to quantitatively evaluate how mutations affect conformational equilibria.

## Supporting Information

Figure S1 **SVD analysis of the chemical shifts measured for the L273W(Apo) mutant and other Wt states depicted in the plot relative to the Rp-cAMPS-bound Wt.** PC1 and PC2 are as explained in the main text. Blue diamonds are the loadings.
(TIF)

Figure S2 **Dendrogram of the largest cluster of residues resulting from the agglomerative cluster analysis of the correlation matrix of** **Figure** **5C** **.** All nodes correspond to Pearson correlation coefficient ≥0.98.
(TIF)
