# Supplementary figures and images for: The Auto-Inhibitory Role of the EPAC Hinge Helix as Mapped by NMR
Source: PLoS One. 2012 Nov 21;7(11):e48707. doi: 10.1371/journal.pone.0048707 (PMC3504058; doi:10.1371/journal.pone.0048707)

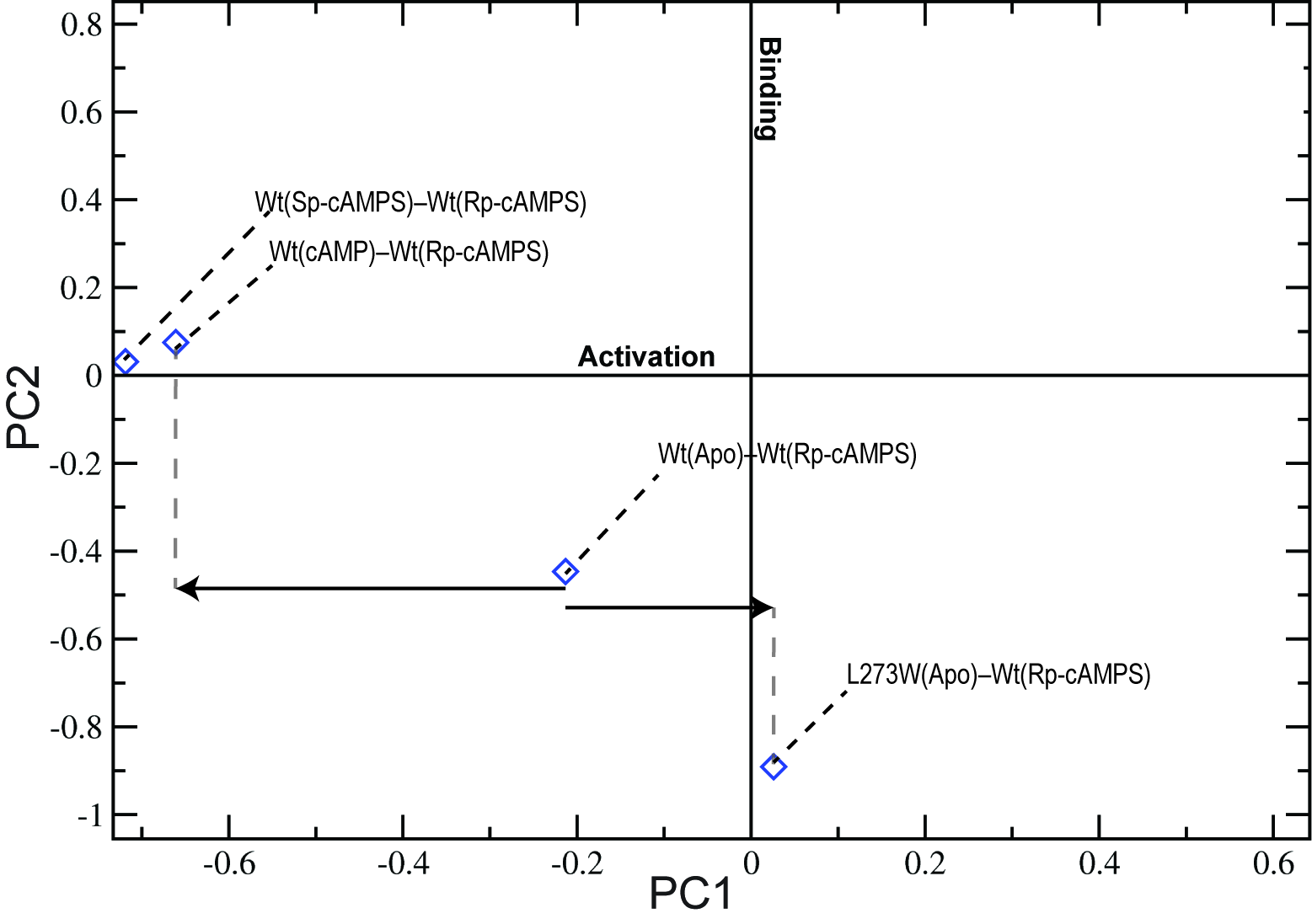

Supplement: Figure S1 — SVD analysis of the chemical shifts measured for the L273W(Apo) mutant and other Wt states depicted in the plot relative to the Rp-cAMPS-bound Wt. PC1 and PC2 are as explained in the main text. Blue diamonds are the loadings. (TIF) [file pone.0048707.s001.tif]

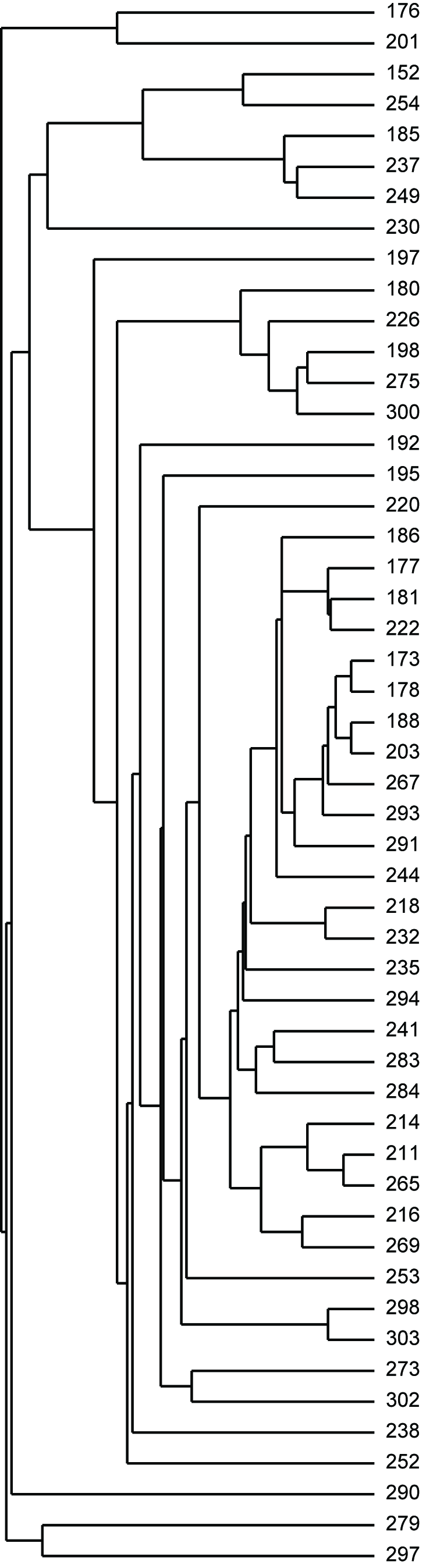

Supplement: Figure S2 — Dendrogram of the largest cluster of residues resulting from the agglomerative cluster analysis of the correlation matrix of Figure 5C . All nodes correspond to Pearson correlation coefficient ≥0.98. (TIF) [file pone.0048707.s002.tif]
